# Social support modulates the neural correlates underlying social exclusion

**DOI:** 10.1093/scan/nsz033

**Published:** 2019-05-05

**Authors:** Rosalba Morese, Claus Lamm, Francesca Marina Bosco, Maria Consuelo Valentini, Giorgia Silani

**Affiliations:** 1 Faculty of Communication Sciences, Università della Svizzera italiana, 6900, Lugano, Switzerland; 2 Social, Cognitive and Affective Neuroscience Unit, Department of Basic Psychological Research and Research Methods, Faculty of Psychology, University of Vienna, 1010, Vienna, Austria; 3 Department of Psychology, research Group on Inferential Processes in Social Interaction - GIPSI, University of Turin, 10124, Turin, Italy; 4 Neuroscience Institute of Turin, University of Turin, 10124, Turin, Italy; 5 Department of Neuroradiology, Hospital-Città della Salute e della Scienza di Torino, 10126, Turin, Italy; 6 Department of Applied Psychology: Health, Development, Enhancement and Intervention, University of Vienna, 1010, Vienna, Austria

**Keywords:** social pain, social support, fMRI, temporal parietal junction, anterior insula

## Abstract

Ostracism threatens the human need for social interactions, with negative consequences on cognition, affect and behavior. Understanding the mechanisms that can alleviate these consequences has therefore become an important research agenda. In this study, we used behavioral and fMRI measures to advance our understanding how social support can buffer the negative effects of social exclusion. We focused on two different types of support from a friend: emotional support, conveyed by gentle touch and appraisal support, implemented as informative text messages. Seventy-one female participants underwent fMRI scanning while playing a virtual ball-tossing game in the course of which they were excluded. Two consecutive runs of the game were separated according to the participant’s experimental condition (appraisal support, emotional support and no support). Results showed that the experience of social exclusion is modulated by the type of support received. Specifically, emotional support decreased negative emotions and anterior insula activity, while appraisal support increased negative emotions, with concomitant increase of subgenual anterior cingulate cortex and decrease of temporal-parietal junction activity. These divergent effects of social support point to the necessity to characterize whether and under which conditions it represents an effective and positive resource to alleviate the negative consequences of social exclusion.

## Introduction

The aim of the present study was to investigate how different types of social support reduce negative feelings associated with social exclusion and its activation at the neural level. Human beings have a fundamental need to interact with each other. Ostracism (social exclusion) threatens this need and has various effects on cognition, affect and behavior (Wesselmann *et al.,* 2012). It is often associated with experiences of pain, often called social pain, defined as ‘the distressing experience arising from the perception of actual or potential psychological distance from close others or a social group’ (Eisenberger and Lieberman, 2004; Eisenberger, 2012). Eisenberger (2012) refers to it as one of the most painful and emotionally unpleasant conditions that the individual can live with, as it bears the risk of damaging his ability to relate to other individuals. Experimental neuroscientific research over the past decades has extensively focused on the understanding of ostracism’s neurophysiological underpinnings. Mainly investigated via computer-controlled ball-tossing games (the cyberball game, see Hartgerink *et al.,* 2015 for review), the experience of exclusion from the game (social exclusion) usually results in feelings of unpleasantness and discomfort, with concomitant recruitment of a network of brain areas associated with the processing of negative affect, such as the dorsal anterior cingulate cortex (dACC), the subgenual anterior cingulate cortex (subACC) (Novembre *et al.,* 2015; Masten *et al.,* 2011a; Masten *et al.,* 2011b; Rotge *et al.,* 2015) and the anterior insula (AI) (Cacioppo *et al.,* 2013). It is currently a matter of debate if the fingerprint of social exclusion resembles the negative experience associated with pain of physical nature (Eisenberger, 2012; Woo *et al.,* 2014). For example, the experiences of social exclusion and physical pain reflect many common psychological and biological characteristics: from the use of similar words (‘I feel hurt’) (Woo *et al.,* 2014), the involvement of overlapping neurochemical (Panksepp *et al.,* 2007; Hsu *et al.,* 2013) and neural systems (Eisenberger, 2012), to comparable inflammatory responses and genetic regulation (Hsu *et al.,* 2013; Hsu *et al.,* 2015; Watkins and Maier, 2000). These commonalities may stem from similar adaptive evolutionary functions (Kurzban and Leary, 2001). As physical damage to an organism threatens its survival, and the presence of pain lead to protective responses via unpleasant and distressing psychological states, feelings of pain and discomfort after separation from the individual’s social group may serve as protective factors preventing such separation. Consequently, social pain may have promoted safety in a similar manner as physical pain; when a ‘socially painful’ event has occurred, it may drive the individual to repair the social relationship or to seek new ones (Sturgeon and Zautra, 2016). However, behaviors that are adaptive when an individual experiences acute pain, e.g. avoiding activities that increase pain, when pain becomes chronic may develop into patterns of behavior that are maladaptive and impair long-term health (Sturgeon and Zautra, 2016). Similarly, social pain responses that are situationally appropriate, e.g. feeling angry or avoiding a group after being rejected, may lead to less-effective coping and long-term social isolation, when they become a chronic issue (Riva *et al.,* 2014). Given the negative and serious long-term consequences of pain exposure, it is mandatory therefore to understand and promote factors that facilitate the remission or prevent the initiation of such psychological and behavioral effects. In that regard, positive aspects of one’s social world (social support) may improve coping responses and overall well-being. For example, according to Shumaker and Brownell (1984, p. 11), social support is configured as an ‘exchange of resources between two individuals, perceived by the one who provides it - or by those who receive it - as something aimed at increasing the well-being of the recipient’. Lin *et al.,* (2011) describes it in terms of perceived and real, useful and/or significant supplies provided by the community, social networks and trustworthy partners associated to the well-being of the subject. House (1981) identifies different types of social support: *`*emotional support’ is associated with sharing life experiences and involves the provision of empathy, love, trust and care; `instrumental support’ involves behaviors that directly help people in need using tangible help (like tangible services and economic benefits); `informational support’ involves the provision of advice, suggestions and information that a person can use to address problems; and finally `appraisal support’ involves the provision of information that is useful for evaluation purposes: constructive feedback, affirmation and social comparison. Several empirical studies (Brown *et al.,* 2003; Younger *et al.,* 2010; Eisenberger *et al.,* 2011) have examined the function of social support on the perception of physical pain, demonstrating a remarkable correlation between social support and the reduction of physical pain experience. Meaningful social connections have also been shown to serve a protective role in reducing neural, physiological and neuroendocrine responses to pain and stress including heart rate, blood pressure and cardiovascular and neuroendocrine responses (Montoya *et al.,* 2004; Ozbay *et al.,* 2007). Given the strong commonalities between physical and social pain, it is not surprising that the interest on the effects of social support on physical pain has been extended to stressors of social nature, with similar results reported. In particular, psychosocial stress caused by social evaluation (Kirschbaum *et al.,* 1993) has been observed to be reduced by social support (Heinrichs *et al.,* 2003). Interestingly, different types of social supports (verbal support, physical contact) have been associated to different reactions in women (Ditzen *et al.,* 2007), suggesting that not all types of social support are effective in reducing the physiological responses to social stress. In spite of the rich scientific literature on social support and psychological stress upon social evaluation, only few studies have directly examined the effects of social support on the feeling of social pain caused by, for example, social exclusion or ostracism. Similarly, to psychological stress, these studies suggest that the presence of a friend (Teng and Chen, 2012), supportive emotional texts (Onoda *et al.,* 2009) or gentle slow touch (von Mohr *et al.,* 2017) are able to reduce the negative feelings caused by social exclusion. On the neural level, self-reported supportive daily life interactions have been shown to diminish neuroendocrine stress responses and to correlate with decreased activity in the dACC following ostracism (Eisenberger *et al.,* 2007). Similarly, Onoda *et al.* (2009) observed that supportive emotional text leads to reduced AI and enhanced theory of mind (ToM) network activity (Saxe and Kanwisher, 2003; Saxe and Wexler, 2005; Schurz *et al.,* 2014; Molenberghs *et al.,* 2016) during social exclusion.

To date, however, a single study examining how different types of social support modulates feelings of social pain and how this is represented at the neural level has not been performed yet. Our study aimed, for the first time, at disclosing the role of different support strategies in modulating the behavioral and neural correlates involved in social exclusion. Specifically, we used two different types of support: emotional physical support (emotional support), which we implemented as gentle touch, and informational/appraisal support (appraisal support), which we implemented as informative text messages allowing to better understand the situation. In line with the previous literature, we hypothesized feelings of social pain, induced via exclusion from a virtual ball-tossing game, to be reduced after experiencing social support. Furthermore, we hypothesized such behavior to be associated with reduced activity of the neural network involved during the experience of social exclusion. Finally, we expect different neurophysiological effects depending on the type of social support experienced. In particular, we expected the emotional support group to show modulatory effect in the affective network (e.g. AI, ACC) while the appraisal support group to additionally modulate the ToM network (Onoda *et al.,* 2009)

## METHODS

### Participants

In total, 81 Italian female volunteers (age, 21.67 ± 2.29 years) with no history of neurological or psychiatric disorders (assessed with semi-structured interviews conducted by a psychologist) were recruited among undergraduate students at the University of Turin. We chose to include females only, as gender differences on social exclusion are well-documented (see Benenson *et al.,* 2013; Tomova *et al.,* 2014). All participants were right-handed according to the Edinburgh Handedness Inventory (Oldfield, 1971). Female friends of a similar age as the participants were invited to participate in the experiment, and instructed to act as confederates. Subjects were randomly assigned to one of the three groups: appraisal support group (N = 26), emotional support group (N = 26) and no support group (N = 29). Ten participants were excluded from the study because of excessive movement or lack of compliance during the functional Magnetic Resonance Imaging (fMRI) session, leaving the final sample for the three groups as follows: appraisal support group (N = 23), emotional support group (N = 23) and no support group (N = 25). All participants signed the information consent after the experimental procedures have been described to them. The study was approved by the Bioethics Committee of the University of Turin.

### Social pain task

**Fig. 1:**
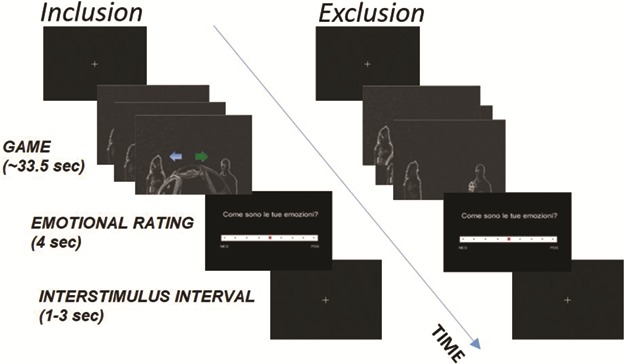
**Exemplar trial for the social pain task.** In each trial, participants played the game with other two virtual players. During the game, once they receive the ball, they have to decide to whom to throw it back (as illustrated by the two arrows) by pressing the left or right key on the pad. In the inclusion condition, participants received the ball at least one third of the total tosses. In the exclusion condition, participants received the ball less than one third of the total tosses. Immediately after the game, they were asked to answer the question ‘How are your emotions?’ on a 9-point Likert scale, displayed for 4 s. Interstimulus interval was randomly jittered between 1 and 3 s. Arrows in the inclusion condition are inserted only for descriptive purposes and not displayed during the game.

In order to create in the fMRI environment the uncomfortable situation in which participants could experience social exclusion, we used a modified version of the well-known `cyberball game’ (Williams *et al.,* 2000), which has been widely used in the literature (Eisenberger and Lieberman, 2004; Masten *et al.,* 2009; Onoda *et al.,* 2009; Bolling *et al.,* 2011; DeWall, 2011). Our version was developed by Novembre *et al.* (2015), who replaced the animated cartoons of the cyberball game by videos showing schematic virtual representations of real people tossing the ball to each other. The task was composed of 10 blocks with two experimental conditions: `social inclusion’ and `social exclusion’. In each block the ball-tossing game included a total of 12 passes, distributed between three players (including the participant). In the five blocks inducing the experience of social inclusion, the participant received at least one third of the total passes, while in the five blocks inducing social exclusion, the participant received less than one third of the total passages (see Novembre *et al.,* 2015 for a detailed description of the stimuli preparation and procedure). Once the participant received the ball, she had to decide to whom to throw it back by pressing with her index (left player) or middle (right player) finger on an Magnetic Resonance Imaging (MRI) compatible button box. The presentation of the blocks was equal for all the participants with a pseudorandomized order: the first three and the last two blocks belonged to the inclusion condition, while the five blocks placed in the central position of the task belonged to the exclusion condition. Each ball-tossing game had an average duration of 33.5 s (range, 30–40 s). At the end of each game, the participant was asked to answer the question ‘How are your emotions?’ in order to report the valence and intensity of the emotions experienced during the game on a Likert scale with nine discrete values (from −4 = very negative on 0 to +4 = very positive) displayed for 4 s. The answer was given by using the same button box used to throw the ball (see Figure 1). Of crucial relevance, this sequence of 10 blocks was performed twice, in two separate fMRI runs. In between the runs, emotional or appraisal support was provided by the participant’s friend in the two experimental groups, while no support was provided in the control group.

### Social support manipulation

Two experimental groups of social support have been defined: emotional and appraisal. In the emotional support group*,* each confederate (the female friend) was instructed to gently touch the hand of the participant, with the aim of comforting her. The characteristic of this group was the administration of support only through physical contact, without the use of verbal or expressive linguistic expressions. No specific constraints on how to deliver the touch was given to the confederates. Rather, they should hold, caress and tenderly squeeze her friend’s hand as she would normally do when trying to comfort her.

In the appraisal support group, social support was given by the participant’s friend through text messages delivered and displayed on the back-projection screen in the scanner. In particular, the participants were told that the phrases they read on the monitor were written and sent directly by their friend from a PC situated in another room, where she could follow the game. Each participant saw 10 pre-prepared phrases meant to give additional information in order to help the understanding of the experience of social exclusion (for example: ‘I think that these two players are actually friends’ or ‘I think that when the experiment will end, we’ll see them go away together’). Importantly, the content of the text was never aimed to comfort the subject but rather to give information allowing the reappraisal of the situation, and it was always emotionally neutral.

Finally, in order to tease apart the effect of the repetition of the task (adaptation, fatigue, etc.), the control group did the social exclusion task twice but without receiving any kind of support in between. We chose such control condition because the mere presence of a friend, even without delivering any social support, could have affected the following experience of social exclusion (Teng and Chen, 2012). All conditions of social support lasted for 3 min and were delivered between runs 1 and 2 of the cyberball game, while the subject was resting inside the scanner. For the no support group, the same interval was kept between runs 1 and 2, and the subject asked to wait still for the next run to start.

### Procedure

**Fig. 2:**
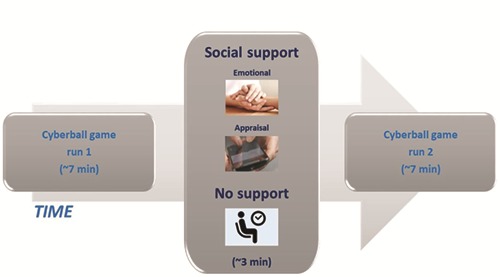
**Timeline of the fMRI session.** Each fMRI session was divided into three phases performed on the same day: (i) social pain task run 1, (ii) social support (emotional, appraisal, no support), (iii) social pain task run 2. Social support was either emotional or appraisal for a duration of 3 min. In the case of the no support group, a 3 min break between the two runs was carried out.

Each participant, previously randomly assigned to one of three groups, and her friend (except for the no support group in which participants came alone) were received in the fMRI room of the hospital and informed about the study. Specifically, participants were told that they would be connected via Internet to two other players, located in another room of the hospital. After the general information, each confederate was accommodated in the adjacent room for observing through a monitor what happens to her friend during the game. Here she was instructed on what she had to do for the different support conditions. For all participants, after the verbal instruction about the cyberball game, a training session was performed outside of the scanner to ensure that participants understood the game. A second short practice session was administered in the scanner to familiarize the participants with the response recording system. The cyberball game was programmed using Cogent toolbox (2000), running on Matlab 2007 (Mathworks, Cherborn, MA, USA). Inside the scanner, the stimuli were presented via a head coil-mounted display system (Resonance Technology, Inc.). The fMRI session was composed of three phases performed on the same day (see Figure 2): (i) social pain task run 1: each participant was scanned while engaging in the virtual cyberball game, as described above; (ii) social support: each experimental group received social support (e.g. emotional or appraisal), while the control group did not receive any kind of support. During this section, no fMRI scanning was performed. (iii) Social pain task run 2: each participant was scanned for the second time while engaging in the virtual cyberball task, as described above. After the fMRI session, each participant answered a brief interview aimed at investigating the believability of the manipulation. In particular, we asked indirect questions such as: ‘What do you think about the players? How was the game for you? Do you have any comments?’ None of the participants expressed doubts about the veracity of the situation.

### MRI data acquisition

The MRI data were acquired using a 3.0 T MRI Scanner (Philips Ingenia) with a 32-channel array head coil. The study was performed at the Center of Brain Imaging 3 T-NIT, at the Hospital Città della Salute e della Scienza in Turin, Italy. Echo-Planar Image (EPI) sequence [TR/TE, 2000/30 ms; 33 slices, matrix size, 64 × 64; interslice gap, 0.5 mm; field of view (FOV), 230 × 230 mm^2^; flip angle, 90 degrees; slices aligned to the AC-PC line, 230 volumes/run] for functional images was applied. A total of 226 volumes per subject per run were collected. The first four volumes of each run were discarded to allow the equilibration of T1 saturation effects. T1-weighted sequence MP-RAGE (TR, 8.1 ms; TI, 900 ms; TE, 3.7 ms; voxel size, 1 × 1 × 1 mm^3^) for structural images of the whole brain was used.

**Table 1:** Contrasts of interest

	**MNI coordinates**			***Z*-score**	***T*-value**	***P*-value**
**Anatomical region**	*X*	*Y*	*Z*			*FWE corrected*
***Emotional support group > no support group***						
*Exclusion run 1 > exclusion run 2*						
Right AI	33	27	−8	3.29	3.35	.052
						
***Appraisal support group > no support group***						
*Exclusion run 1 > exclusion run 2*						
rTPJ	46	−47	27	3.42	3.48	.046
lTPJ	−48	−53	34	3.22	3.27	.001^*^
						
***Appraisal support group > no support group***						
*Exclusion run 2 > exclusion run 1*						
Left subACC	−5	32	−5	3.34	3.39	.046
Right vmPFC	2	37	−8	2.99	3.03	.001^*^

Significant voxels are reported threshold of *P* ≤ 0.05 FWE corrected for small volumes.Peak activity coordinates are given in MNI space.^*^Significant value for *P* < 0.001 uncorrected.

### Data analysis

#### Behavioral analysis

Emotional ratings given by the participants after each round of the cyberball game were analysed in order to investigate differences in the emotional experience between exclusion and inclusion trials and between the first and second run, i.e. before and after receiving social support. We conducted a repeated-measures ANOVA with two within-subjects factors, condition (inclusion, exclusion), time (runs 1 and 2), and one between-subject factor, group (emotional support, appraisal support, no support). Ratings of the exclusion condition were multiplied by −1 in order to carry the same direction as the inclusion ratings, allowing to test the three-way interaction. Statistical analyses were performed with IBM SPSS Statistics version 24.

#### fMRI data analysis

The MRI data were analysed using Statistical Parametric Mapping 12 (SPM12, Wellcome Department of Cognitive Neurology, London, UK) run on Matlab 2007 (Mathworks, Cherborn, MA, USA). All functional images have been pre-processed following this order: spatially realigned to the first volume, co-registered to the mean image, segmented in cerebrospinal fluid tissues, gray matter and white matter, then normalized to the Montreal Neurological Institute (MNI) space and finally smoothed at the first level with an 8 mm full-width half-maximum Gaussian Kernel, with an additional 6 mm at the second level. Low-frequency drifts, high-pass temporal filtering with a cut-off of 128 s was used. After preprocessing, a General Linear Model (Friston *et al.*, 1995) for statistical analysis was used for both functional runs. Regressors of interest were convolved with a canonical hemodynamic response function. For each participant’s first level analysis, six regressors were computed: social inclusion (I), social exclusion (I), emotion rating (I), social inclusion (II), social exclusion (II) and emotion rating (II). In addition, six parametric regressors of no interest were added to the design matrix to correct residual effects of head motion. At the second level, four contrasts of interest from the first-level analyses were fed into a flexible factorial design aiming at investigating the effect of social support on social exclusion, using a random effects analysis (Penny, Holmes and Friston, 2003). Linear contrast of the repeated-measures ANOVA with the within-subject factors, condition (exclusion, inclusion), time (runs 1 and 2), and the between-subject factor, group (emotional support, appraisal support, no support), were used to assess the interaction between the factors group and time. Given the main research question of our paper, only results for the exclusion condition are reported. We performed whole brain analyses with an initial threshold of *P* < 0.001 uncorrected and reports clusters that survived Family-Wise Error (FWE) correction for small volumes (SVC) at *P* < 0.05. For the SVC, we created two binary masks encompassing, first, the affective network specifically detected in social exclusion paradigms, and second, a network associated to representing other minds and intentions (ToM). Both masks are based on the most recent published meta-analyses on social exclusion and ToM, respectively. More specifically, the first mask included coordinates derived from two meta-analyses on social exclusion published by Cacioppo *et al.*, 2013 and Rotge *et al.*, 2015. In spite of repeated attempts, it was, however, not possible to receive the original maps from both authors. Therefore, spheres of 10 mm radius centered on the reported main activation loci were generated and combined into one mask with the toolbox MarsBaR (Brett *et al.,* 2002). The second mask was provided as an image-based mask by (Molenberghs et al., 2016), based on their meta-analysis on ToM tasks (see supplementary materials for more details). Given we did not expect the involvement of the ToM network for the emotional support group, only the first (affective) mask was used to investigate differences in activations between this group and the no support group. To investigate differences in activations between the appraisal support and no support groups and the emotional support and appraisal support groups, both the affective and the ToM masks were used. The MRIcron software package (Rorden *et al.,* 2007a; Rorden *et al.,* 2007b) was used for anatomical and cytoarchitectonic display and interpretation.

#### Brain–behavior correlation analyses

Pearson correlation analyses between brain activity and behavioral ratings were performed with IBM SPSS Statistics version 24. In particular, the difference in activity (∆) between the first and second run of social exclusion in the regions showing significant statistical difference (see Table 1) was correlated with the difference in emotional ratings between the two runs (run 1 minus run 2). Activity in these regions was extracted with REX (http://web.mit.edu/swg/rex/rex.pdf). Correlations were performed for each group separately and corrected for the number of ROIs used in each group.

## RESULTS

### Behavioral results

**Fig. 3:**
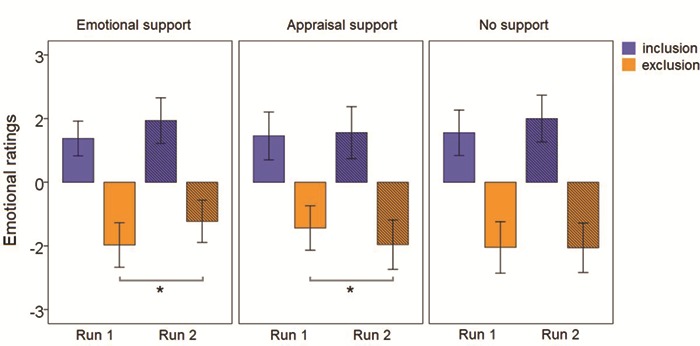
**Behavioral results.** Mean and confidence intervals (95%) divided by group, condition and run. Significant differences are marked with an asterisk (*P* < 0.05, based on post hoc pairwise comparisons)

The ANOVA revealed a significant interaction effect time*condition*group [*F*_(2,68)_ = 3.39, *P* = 0.040, partial Eta squared = 0.091]. All the other effects and interactions were not significant (*F* < .103)^1^. Post hoc pairwise comparisons were used in order to characterize the effect of the triple interaction. In particular, in the emotional support group, a significant difference between exclusion run 1 *vs* run 2 was observed, defined by a reduction of unpleasantness ratings during the second run (*M*_diff_ = 5.57, SE = 1.91, *P* = 0.005). In the appraisal support group*,* a significant difference between exclusion run 1 *vs* run 2 was also observed, but with an opposite pattern, namely an increase of unpleasant emotions in the second run (*M*_diff_ = −3.93, SE = 1.91, *P* = 0.044). The no support group did not show any significant difference between runs 1 and 2 for both conditions (see Figure 3). Finally, the differences between the inclusion and exclusion runs (Δ inclusion, Δ exclusion) were entered in a one-way ANOVA to assess whether the groups significantly differed. The analysis revealed a significant difference between the groups in the Δ exclusion only (*F*_(2,68)_ = 6.22, *P* = 0.003). Post hoc multiple comparisons were used in order to characterize the effect. In particular, we observed a significant difference both between the emotional support group and the no support group (*M*_diff_ = 0.564, SE = 0.265, *P* = 0.037) and the emotional support group and the appraisal support group (*M*_diff_ = 0.949, SE = 0.270, *P* = 0.001).

### fMRI results

***Emotional support group vs. No support group.***

*Emotional support (social exclusion run 1 > social exclusion run 2) > no support (social exclusion run 1 > social exclusion run 2).*

**Fig. 4:**
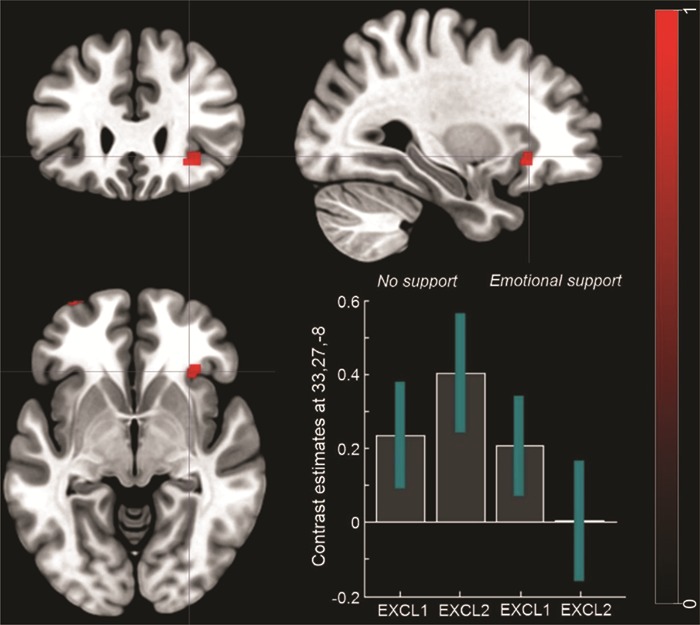
**FMRI results.** Differences in the neural activation between the emotional support group vs. the no support group for the contrast: social exclusion run 1 *>* social exclusion run 2. The bar plots represent contrast estimates and 90% confidence intervals in the right AI. For illustrative purposes, statistical maps are displayed with a threshold of *P* < 0.001 uncorrected and superimposed on a standard T1 template.

The analysis revealed significantly reduced activation in the right AI (rAI, x = 33, y = 27, z = −8) for the emotional support group compared to the no support group (Figure 4; Table 1) for the second run compared to the first run of social exclusion^2^.

*Emotional support (social exclusion run 2 > social exclusion run 1) > No support (social exclusion run 2 > social exclusion run 1).*

No suprathreshold voxels were observed for the reverse contrast.

***Appraisal support group vs. no support group.***

*Appraisal support (social exclusion run 1 > social exclusion run 2) > No support (social exclusion run 1 > social exclusion run 2).*

**Fig. 5:**
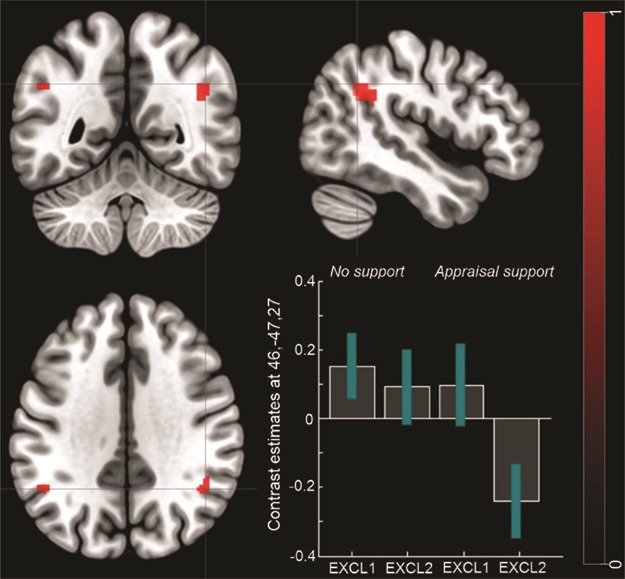
**FMRI results.** Differences in the neural activation between the appraisal support group *vs* the no support group for the contrast: social exclusion run 1 > social exclusion run 2. The bar plots represent contrast estimates and 90% confidence intervals in the right TPJ. For illustrative purposes, statistical maps are displayed with a threshold of *P* < 0.001 uncorrected and superimposed on a standard T1 template.

The analysis revealed significantly reduced activation in the right temporal parietal junction (rTPJ, x = 46, y = −47, z = 27) for the appraisal support group compared to the no support group (Figure 5; Table 1) for the second compared to the first run of social exclusion. A more liberal threshold of *P* < 0.001 revealed reduced activation also in the left temporal parietal junction (lTPJ, x = −48, y = −53, z = 34).

*Appraisal support (social exclusion run 2 > social exclusion run 1) > No support (social exclusion run 2 > social exclusion run 1).*

**Fig. 6:**
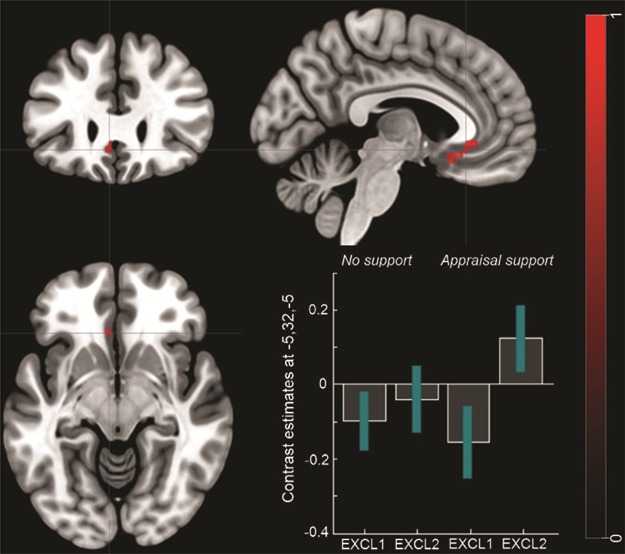
**FMRI results.** Differences in the neural activation between the appraisal support group *vs* the no support group for the contrast: social exclusion run 2 > social exclusion run 1. The bar plots represent contrast estimates and 90% confidence intervals in the subACC. For illustrative purposes, statistical maps are displayed with a threshold of *P* < 0.001 uncorrected and superimposed on a standard T1 template.

The analysis revealed significantly increased activation in the subACC (x = −5; y = 32, z = −5) for the appraisal support group compared to the no support group (Figure 6. Table 1) for the second compared to the first run of social exclusion. A more liberal threshold of *P* < 0.001 revealed reduced activation also in the ventromedial prefrontal cortex (vmPFC) (2, y = 37, z = −8).

**Fig. 7:**
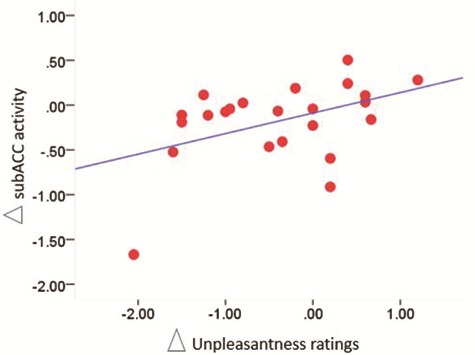
**Correlation results.** Scatterplot of the correlation between the difference in subACC activity between exclusion runs 1 and 2 (∆ subACC) and the difference in unpleasantness ratings between exclusion runs 1 and 2.

***Emotional support group vs. Appraisal support group.***

No suprathreshold voxels were observed in any of the possible combinations.

### Brain-behavior correlation analyses

The following correlations were performed: (i) for the emotional support group, correlation between ∆ activity in rAI and ∆ unpleasantness ratings and (ii) for the appraisal support group, correlation between ∆ activity in rTPJ, subACC and ∆ unpleasantness ratings. The correlation analyses revealed a significant positive relationship between ∆ subACC and ∆ unpleasantness ratings in the appraisal support group (r(23) = 0.443, *P* < 0.017 one tailed, corrected for the number of correlations performed). This means that in the appraisal support group, the increase of subACC activity observed in the second run of exclusion was associated to increased unpleasantness feelings in the second run (Figure 7). All the other correlations were not significant.

## DISCUSSION

In the present study, we investigated the effects of different types of social support (emotional and appraisal) on the behavioral and neural correlates of the experience of social exclusion. Seventy-one female participants were scanned twice while playing the cyberball game. Between the two runs of the game, different types of support were delivered by a female friend. At the behavioral level, we observed that, compared to the control group (no support), the sample that received emotional support in the form of gentle touch, reported reduced feeling of unpleasantness during exclusion trials between the first and second run of the game, i.e. after they had received the emotional support. Our results are in line with the findings of von Mohr *et al.* (2017), which showed reduced reported distress associated to ostracism, after being touched with optimal speed (3 cm/s) to induce positive feelings and thereby promoting interpersonal touch and affiliative behavior (McGlone *et al.,* 2014). By adding these results, our study was able to show for the first time that the experience of emotional support is associated, at the neural level, to a reduction of activity in right AI, a brain area involved in the processing of negative affect during social exclusion and self- and other-directed aversive experiences (Eisenberger *et al.,* 2003; Lieberman *et al.,* 2004; Singer and Lamm, 2009). The effects of emotional social support on the experience of social pain resemble the findings reported on pain of physical nature (Coan *et al.,* 2006; Younger *et al.,* 2010). In particular, during the administration of painful stimuli, married women who held the hand of their partners indicated a lower value of perceived pain. The subjective experience was correlated with reduced activation of the brain areas involved in pain processing, including the AI (Coan *et al.,* 2013). Moreover, imagined social support, provided through the visualization of images portraying of loved ones, was also able to modify the neural activation of the insula (Younger *et al.,* 2010; Eisenberger *et al.,* 2011) and reduce the feeling of distress upon physical pain. The similar effect of emotional support on social and physical pain suggests overlapping regulatory mechanisms, possibly associated to the activity of the μ-opioid system and its analgesic properties (Nummenmaa *et al.,* 2016).

The more informative type of support yielded instead different results. At the behavioral level, participants reported increased feelings of unpleasantness after receiving information about the other two participants. The subjective experience was accompanied by a reduced activation in the right TPJ, an area included in the ToM network (Saxe and Kanwisher, 2003; Saxe and Wexler, 2005; Saxe and Powell, 2006) and involved in incongruency detection and self-other distinction (van Overwalle, 2008; Lamm *et al.,* 2016; Soutschek *et al.,* 2016). TPJ is considered a central structure implicated in the representation of mental states of others (Saxe and Kanwisher, 2003). A recent study has associated the function of this brain region to the update of the internal models of the situation in order to generate appropriate actions to the social contexts (Geng and Vossel, 2013). This function is particularly important when faced with unexpected stimuli that demand attention reorienting and model updates. The findings of the present study suggest that the information received during the support possibly allowed the participants to interpret what was happening during the first run of the game. Indeed, the participants that received information (e.g. ‘the two players are friends’ or ‘there is understanding between them’) leading to a better understating of the social situation, showed an increase of unpleasant emotions (possibly anger) and possibly a reduced need to understand what was happening, indicated by reduced activity in TPJ. To corroborate this hypothesis, we observed increased recruitment of the subACC after receiving the appraisal support. Furthermore, the increased activity in subACC was positively correlated with the increased negative feelings reported during the second run. Interestingly, the subACC is a region involved in affective processes but not in physical pain (Devinsky *et al.,* 1995). Several social pain studies have indicated an increase in activity in the subACC during the negative experience of social exclusion (Bolling *et al.,* 2011; Onoda *et al.,* 2010; Novembre *et al.,* 2015). Masten *et al.,* (2011a) indicated the possibility that greater responsivity in the subACC during peer rejection could reflect an inability to properly regulate emotions evocated by negative events. In line with this literature, some studies have shown that this area is more responsive to negative emotional stimuli among depressed patients and correlates to the severity of depressive symptoms (Teng and Chen, 2012; Davidson, Irwin, Anderle, and Kalin, 2003). Notably and differently from subACC, the increased negative affect did not result in a concomitant increase of AI activity, suggesting that the effects of ostracism on affective pain-related brain areas were not modulated by this type of support received. These findings point to a different role of these two areas in emotional processing during social exclusion, possible link to affective saliency and the need of emotion regulation. Our results are partially in line with the findings by Ditzen *et al.* (2007), who reported different reactions depending on the type of social support (verbal or physical contact) received. In particular, they observed that only physical contact was effective in reducing the symptoms of distress associated to negative social evaluation, while verbal support did not show any different from the no support condition. In our case, though, the appraisal support group showed increased negative feelings and concomitant neural response. It is possible to speculate that the negative reaction observed after appraisal support could have adaptive functions for the person experiencing it, in that it may drive the individual to seek for new relationship, when the actual ones are dysfunctional (Sturgeon and Zautra, 2016).

In conclusion, our study provides the first neuroimaging evidences that experiences of social support can modulate regions of the brain recruited during social pain and possibly responsible for coding the negative valence and intensity of emotion experience. Furthermore, for the first time, we showed that this effect may be different depending on the type of support received. Social support is a very complex phenomenon in which various factors can influence how it is effective for the receiver (e.g. who is providing it, in which form, etc.). It has been shown that it does not always result in a reduction of the negative experiences associated to social stress (Ditzen *et al.,* 2007) and social pain. Instead, as observed in our study, it can also increase the negative emotional experience, which can still be functional for the individual in the short term. Therefore, it is very important to understand under which conditions (contextual, personal, modality, etc.) social support can represent an effective and positive resource to alleviate the negative consequences of social exclusion. Importantly, our findings are restricted to a female sample; therefore not generalizable to the entire population. Future studied are needed to extend these findings to samples representative of the general population such as male participants and different age groups (Riva *et al.,* 2018) and to explore alternative types of social support (e.g. instrumental, informational).

## Supplementary Material

scan-18-352-File003_nsz033

## Footnotes

1 Note that due to the reversing of the sign of the ratings of the exclusion condition, the ANOVA is testing for difference in intensity and not valence. If not reversed, a significant main effect of condition is observed (*F*_(1,68)_ = 179.95, *P* = 0.000, partial Eta squared = 0.726)

2 Left anterior insula (lAI, x = −34, y = 25, z = −12) only survived a more liberal threshold of *P* = 0.003 uncorrected
