# Supplementary material for: Social support modulates the neural correlates underlying social exclusion
Source: Soc Cogn Affect Neurosci. 2019 May 5;14(6):633–43. doi: 10.1093/scan/nsz033 (PMC6688450; doi:10.1093/scan/nsz033)
Supplement: scan-18-352-File003_nsz033 [file scan-18-352-file003_nsz033.docx]

**Supplementary Materials**

***Affective mask***

The affective mask included coordinates derived from previous meta-analyses on social pain (Cacioppo et al., 2013; Rotge et al., 2015). The mask encompasses bilateral anterior insula (38 18 -6, -36 20 -10), mid cingulate cortex (8  22  26), anterior cingulate cortex (-2 52 10), subACC (4 36 -4) and left inferior orbito-frontal lobe (-32 16 -20). Spheres of 10 mm radius centered on the activation loci were generated and combined with the toolbox MarsBaR (Brett et al. 2002). Total volume(mm)=23776.


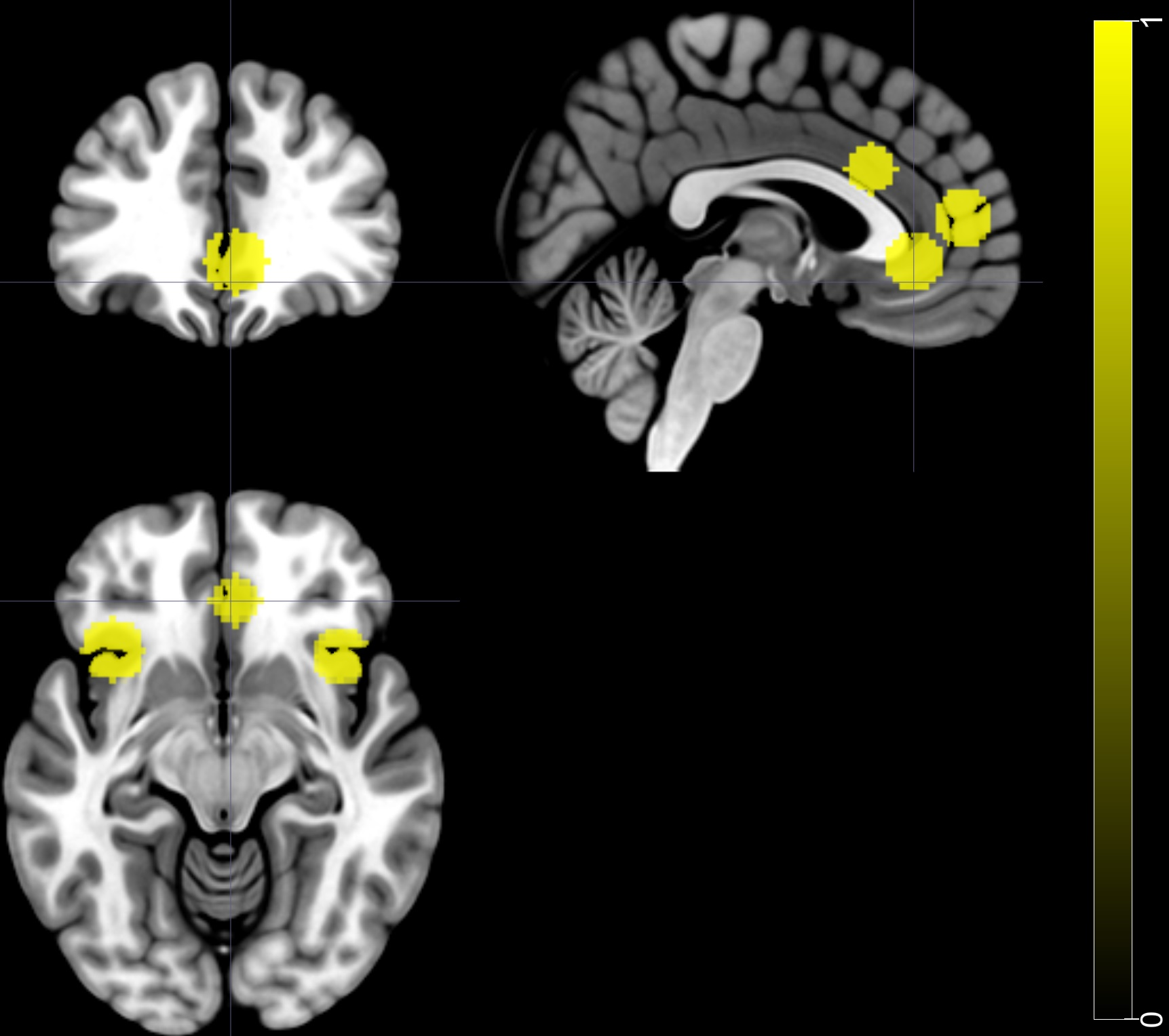


***Figure S1.*** The affective mask superimposed on a standard T1 template

***ToM mask***

The ToM mask was provided by Molenberghs and colleagues (Molenberghs  et al. 2016). It encompasses medial frontal cortex, bilateral temporo-parietal junction, bilateral temporal sulcus, bilateral temporal poles and precuneus. Total volume(mm)=23776.


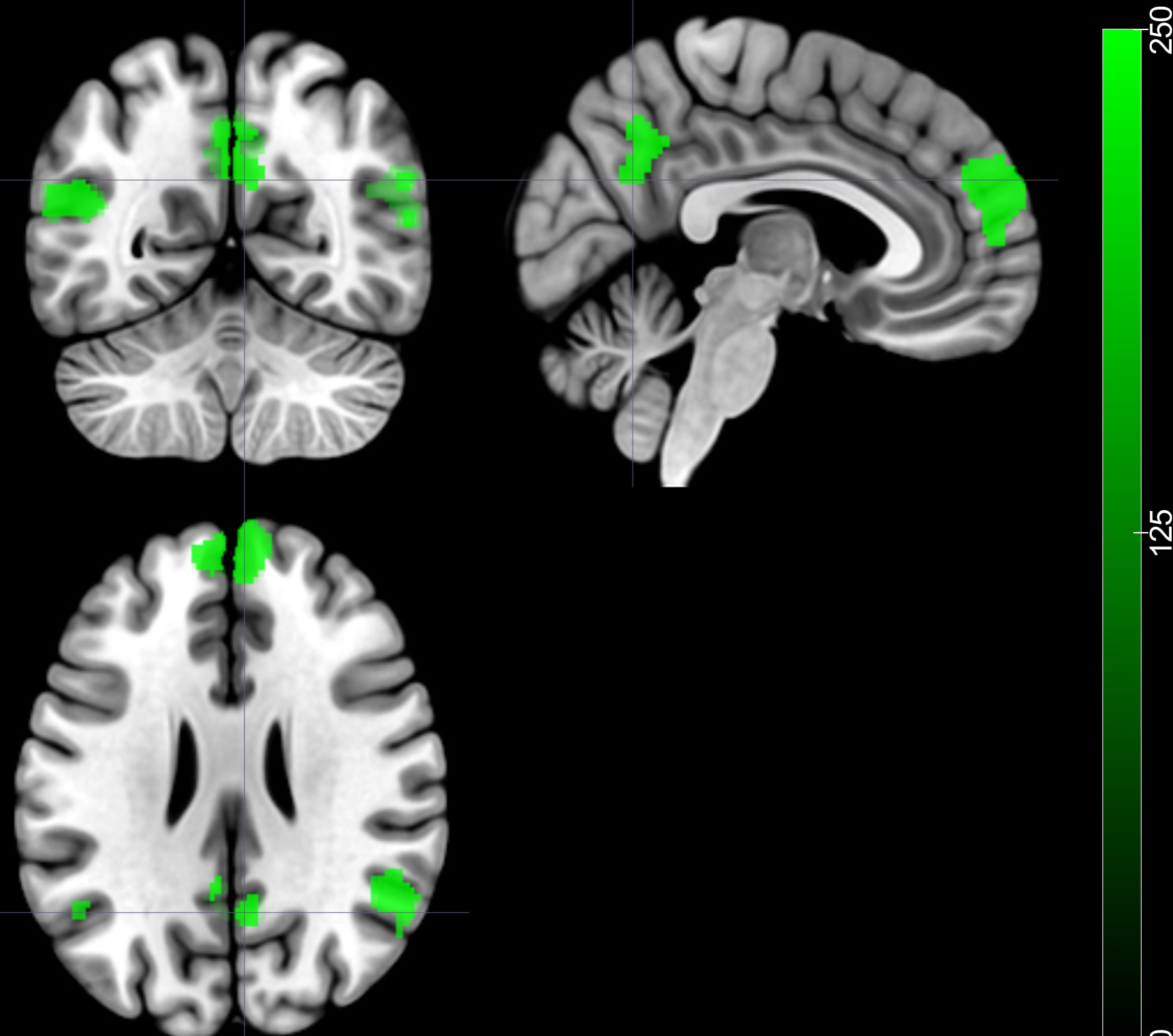


***Figure S2.*** The ToM mask superimposed on a standard T1 template

**References**

Brett, M., Anton J.L, Valabregue, R., Poline, J.B. Region of interest analysis using an SPM toolbox. Presented at the 8th International Conferance on Functional Mapping of the Human Brain, June 2-6, 2002, Sendai, Japan. Available on CD-ROM in NeuroImage, Vol 16, No 2, abstract 497.

Cacioppo, S., Frum, C., Asp, E., Weiss, R. M., Lewis, J. W., & Cacioppo, J. T. (2013). A quantitative meta-analysis of functional imaging studies of social rejection. *Sci Rep, 3*, 2027. doi:10.1038/srep02027.

Molenberghs, P., Johnson, H., Henry, JD. (2016) Understanding the minds of others: A neuroimaging meta-analysis. *Neuroscience and Biobehavioral Reviews* 65: 276–291.

Rotge, J. Y., Lemogne, C., Hinfray, S., Huguet, P., Grynszpan, O., Tartour, E., et al. (2015). A meta-analysis of the anterior cingulate contribution to social pain. Soc. Cogn. Affect. Neurosci. 10, 19–27. doi: 10.1093/scan/nsu110.
